# CAPABLE Program Improves Disability in Research and Implementation Settings

**DOI:** 10.1093/geroni/igab046.449

**Published:** 2021-12-17

**Authors:** Sarah Szanton, Qiwei Li, Laura Gitlin

**Affiliations:** 1 Johns Hopkins University, Baltimore, Maryland, United States; 2 Johns Hopkins School of Nursing, Baltimore, Maryland, United States; 3 Drexel University, College of Nursing and Health Professions, Drexel University, Pennsylvania, United States

## Abstract

Interventions to reduce disability are crucial for older adults with disabilities to avert unnecessary hospitalizations or nursing home placements and improve daily life. Developed and tested at one research site, multiple health systems and community based organizations have since implemented CAPABLE. All published or peer reviewed tests of CAPABLE were reviewed (six studies, 11 sites) with a total of 1087 low-income community-dwelling older adults with disabilities. Participants were an average age of 74-79, cognitively intact, and self-reported difficulty with one or more activities of daily living (ADL). These trials were reviewed by extracting the participants’ scores on main outcomes, ADLs and IADLs, and when available, fall efficacy, depression, pain and cost savings. All studies yielded improvements in ADL and IADL limitations, with small to strong effect sizes. Studies with the complete dose of CAPABLE showed more improvement in ADLs and cost savings than the studies that implemented a decreased dose.

